# Reversed brain size sexual dimorphism accompanies loss of parental care in white sticklebacks

**DOI:** 10.1002/ece3.1175

**Published:** 2014-07-27

**Authors:** Kieran Samuk, Davis Iritani, Dolph Schluter

**Affiliations:** Department of Zoology and Biodiversity Research Centre, University of British ColumbiaVancouver, British Columbia, Canada

**Keywords:** Adaptation, brain size, fish, Parental care, stickleback

## Abstract

Uncovering factors that shape variation in brain morphology remains a major challenge in evolutionary biology. Recently, it has been shown that brain size is positively associated with level of parental care behavior in various taxa. One explanation for this pattern is that the cognitive demands of performing complex parental care may require increased brain size. This idea is known as the parental brain hypothesis (PBH). We set out to test the predictions of this hypothesis in wild populations of threespine stickleback (*Gasterosteus aculeatus*). These fish are commonly known to exhibit (1) uniparental male care and (2) sexual dimorphism in brain size (males>females). To test the PBH, we took advantage of the existence of closely related populations of stickleback that display variation in parental care behavior: common marine threespine sticklebacks (uniparental male care) and white threespine sticklebacks (no care). To begin, we quantified genetic differentiation among two common populations and three white populations from Nova Scotia. We found overall low differentiation among populations, although *F*_ST_ was increased in between-type comparisons. We then measured the brain weights of males and females from all five populations along with two additional common populations from British Columbia. We found that sexual dimorphism in brain size is reversed in white stickleback populations: males have smaller brains than females. Thus, while several alternatives need to be ruled out, the PBH appears to be a reasonable explanation for sexual dimorphism in brain size in threespine sticklebacks.

## Introduction

Vertebrates have long been known to display impressive levels of variation in the size and shape of their brains. Yet, the evolutionary and proximate forces that shape this variation remain poorly understood. Brain morphology (overall size and the size of individual structures) has been shown to correlate with diverse behavioral, sensory, and ecological variables across a wide variety of taxa (Gittleman 1994; Lefebvre et al. 1997; Farris 2005; Gonda et al. 2009; Smith et al. 2010). However, establishing specific associations between neurological traits and other organismal traits remains a major challenge for evolutionary biologists.

One specific behavioral trait recently suggested to shape variation in brain morphology is parental care (Gonzalez-Voyer et al. 2009a). Parental care often involves a wide variety of complex and novel behavioral interactions between parent and offspring (Clutton Brock 1991). Because increased behavioral complexity likely requires increased neurological complexity (Lefebvre et al. 1997, 2004; Kotrschal et al. 1998; Reader and Laland 2002; Lindenfors 2005), we might expect the evolution of parental care to require concomitant evolution of brain structures involved in performing care behaviors. Further, the degree of complexity of these behaviors should correspond to the degree of brain elaboration. This idea is known as the *parental brain hypothesis* (herein PBH; Dunbar 1998, Gonzalez-Voyer et al. 2009a).

While intriguing, this hypothesis has received limited attention. Thus far, the best evidence comes from macroevolutionary studies of two groups, mammalian carnivores and cichlids, where it has been shown that females of maternally caring species have larger brains than females of biparental species (Gittleman 1994; Gonzalez-Voyer et al. 2009a). These two studies used phylogenetically independent contrasts to analyze the relationship between brain size and parental care, while statistically controlling for confounding factors. For example, Gonzalez-Voyer et al. (2009a) showed that a correlation between brain size and parental care in cichlids is robust to variation in habitat and diet.

Along with macroevolutionary studies, intraspecific or interpopulation studies can provide a test-bed for hypothesis such as of the PBH (Gonda et al. 2013). Such an approach has been applied to testing other correlates of brain morphology both halictid bees and ninespined sticklebacks (Gonda et al. 2009;, Smith et al. 2010). Interpopulation studies have several key benefits, such as disentangling selection versus drift (e.g., *Q*_ST_ vs. *F*_ST_) and opening the door to genetic dissection of trait associations (e.g. via QTL from interpopulation crosses, Gonda et al. 2013).

One system where interpopulation comparisons may yield insight into the PBH is threespine sticklebacks (*Gasterosteus aculeatus*). These fish have long been known to exhibit male uniparental care (Hancock 1852; Tinbergen 1951; Wootton 1984). Intriguingly, Kotrschal et al. (2012) recently found that male threespine sticklebacks also have larger brains by weight than females in two Icelandic populations (also reported in European sticklebacks by Titschack 1921). They speculated that this difference might reflect the increased cognitive demands placed on males by courtship and/or parental care experienced by males (i.e., the PBH). However, these authors identified two limitations in their dataset: a lack of population-level replication and an absence of stickleback populations exhibiting variation in parental care behavior (Kotrschal et al. 2012).

In this study, we sought to expand on these results by comparing brain size between sexes in seven stickleback populations from Canada that vary in parental care phenotype. Four of these populations are marine threespine stickleback populations, two from British Columbia and two from Nova Scotia, are thought to exhibit the standard paternal care phenotype (Pressley 1981; Bell & Foster 1994). The remaining three are populations of a unique “white” form of threespine stickleback found in Nova Scotia (Blouw and Hagen 1990). White sticklebacks differ from the common threespine stickleback in several ways: they are smaller; they generally nest in beds of filamentous algae; males have white nuptial coloration; and, most importantly, male white sticklebacks do not perform any parental care behavior (Jamieson et al. 1992a; Blouw 1996). In spite of these substantial differences, white sticklebacks likely diverged from common sticklebacks recently (Haglund et al. 1990).

Given these characteristics, the loss of parental care in white sticklebacks provides a unique opportunity to test the PBH. To our knowledge, the PBH has not yet been tested in a system where parental care has been lost or reduced from an ancestral state. If larger brains are required to perform parental care (and are metabolically expensive to maintain otherwise), the PBH predicts that the loss of parental care in the white sticklebacks should be accompanied by a corresponding reduction in male brain size. We would expect this to be reflected in both a change in sexual dimorphism within white sticklebacks (male brains no longer larger than female brains), as well as a reduction in male brain size relative to the ancestral (i.e., common) state. We tested these ideas by comparing male and female brain size in multiple natural populations of white and common sticklebacks. To provide evolutionary context for the measurements of brain size, we also estimated the genetic relationship among populations of stickleback in Nova Scotia.

## Materials and Methods

### Collections

We collected white sticklebacks at three sites in Nova Scotia, and common sticklebacks from two sites in Nova Scotia and one site in British Columbia (See Table S1 for collection site details) in early June 2012. We chose these sites based on the presence of breeding males defending nests. This was performed to enrich our sample for adult fish, as well as to aid in identifying white sticklebacks (males are only white during the breeding season, Blouw and Hagen 1990). We also examined common sticklebacks collected from an additional site in British Columbia in 2008. We caught fish by deploying minnow traps approximately 5–15 m from the shore and retrieving them 3 h later. Upon capture, we euthanized all fish via an overdose of Finquel MS-222 (Argent Laboratories, WA) and preserved them in 95% ethanol. In total, our sample contained 162 common sticklebacks and 90 white sticklebacks from the seven locations (Table S1).

### Genotyping and *F*_ST_ calculation

To assess the genetic relationship among the populations in our study, we genotyped 16 individuals from each of the five Nova Scotia populations using GBS (genotyping by sequencing, Elshire et al. 2011). After library preparation using the restriction enzyme *PstI* (New England Biolabs, Ipswich), the resultant GBS library was sequenced using an Illumina HiSeq 2000 (Illumina, San Diego). We aligned reads to the threespine stickleback reference genome (Jones et al. 2012; obtained from Ensembl) and called SNPs using the Unified Genotyper in GATK (McKenna et al. 2010). We filtered called SNPs for quality using the function SelectVariants in GATK with the following filter expression: QD < 2.0 || FS > 60.0 || MQ < 40.0 || HaplotypeScore > 13.0 || MappingQualityRankSum < −12.5 || ReadPosRankSum < −8.0 (filter obtained from GATK Best Practices Document, http://www.broadinstitute.org/gatk/guide/best-practices). This resulted in a dataset containing 12,667 high-quality SNPs. We then carried out calculation of pairwise FSTs using the pairwise.fst() function in the R package “adegenet” (Jombart 2008). We did not analyze the relationship between Pacific and Atlantic sticklebacks – however, they are estimated to have been isolated from the Atlantic populations for 90–260 thousand years (Orti et al. 1994).

### Measurements and dissections

We carried out measurements and dissections of all specimens during May–July 2013. We first took lateral photographs of all specimens (along with a standard ruler) using a digital camera. Next, we determined the sex of all individuals by examining nuptial coloration and gravidity. When sex of a specimen was ambiguous, we confirmed it by making an abdominal incision and directly inspecting the gonads. Finally, we measured standard length for all fish using imageJ (Rasband 1997). We did not attempt to size-match individuals within or across populations for inclusion in the study, and thus, we ultimately dissected all the individuals we collected (see Fig. S1 for body size distributions).

To remove brains from our specimens, we began by laterally bisecting the skull between eyes. We then made a medial incision along the entire skull from between the eyes to the back of the head. Further cuts were made from this medial incision to expose the brain. We then excised the optic nerves and removed the brain from the brain case. The small size of white sticklebacks coupled with the dehydrating effects of ethanol on brain tissue prevented us from consistently recovering the whole hindbrain from all specimens. Hence, we excised it from the brain of all our specimens by making a cut at the hindbrain–cerebellum interface. We stored all brains in 95% ethanol inside 1.5-mL centrifuge tubes for 24 h prior to weighing.

We weighed brains using a XP6U microbalance (Mettler-Toledo, OH). Before weighing, we removed each of the brains from their storage tube and placed them briefly on a piece of filter paper to remove excess fluid. We then weighed the brains in a small foil weigh boat. We performed three serial measurements of weight for each brain and averaged these for use in our analyses. To confirm that incompletely desiccating the brains did not introduce bias in our dataset, we also dried a subset of the brains (10 males and 10 females each from one population of each type) in an incubator for 48 h at 55°C, and weighed them as before. The completely desiccated subset of brain mirrored all the patterns we found (Fig. S2), and there was a strong, significant correlation between dry and wet weight (*r* = 0.88, *t* = 8.69, df = 33, *P* ≪ 0.001).

### Analyses

To test the hypothesis that white sticklebacks have altered sexual dimorphism in brain weight, we fit a linear mixed model to our brain size data using the R package *nlme (*Pinheiro et al. 2013). Because brain size is known to scale with body size (Brandstätter and Kotrschal 1990), we included standard length as a covariate. We also applied a logarithmic transformation to our brain weight data in order to normalize regression residuals and included sampling locality as a random effect in the model. The final model had the following form: log cube-root brain weight = log standard length + sex (male, female) + type (white, common) + sex*type (interaction) + standard length*type + intercept + population (random intercept). To probe the above model, we also fit separate models for each sex, with the following form: log cube-root brain weight = log standard length + type + standard length*type + intercept + population (random).

Finally, we asked whether male common sticklebacks on average have larger brains than female common sticklebacks, that is, whether the result reported in Kotrschal et al. (2012) is also true of Canadian populations. To do this, we fit another linear mixed model to only the common stickleback data. This model had the following form: log cube-root brain weight = standard length + sex (male or female) + intercept + population (random intercept).

## Results

### Genetic relationships

We found that genomewide genetic differentiation between all Nova Scotian populations was overall very small – the average *F*_ST_ between all populations was 0.027. However, *F*_ST_ values were increased between types (0.0307: white vs. common) compared with within types (0.0231: common vs. common, 0.0222 white vs. white) (Table 2).

### Brain size dimorphism

**Figure 1:**
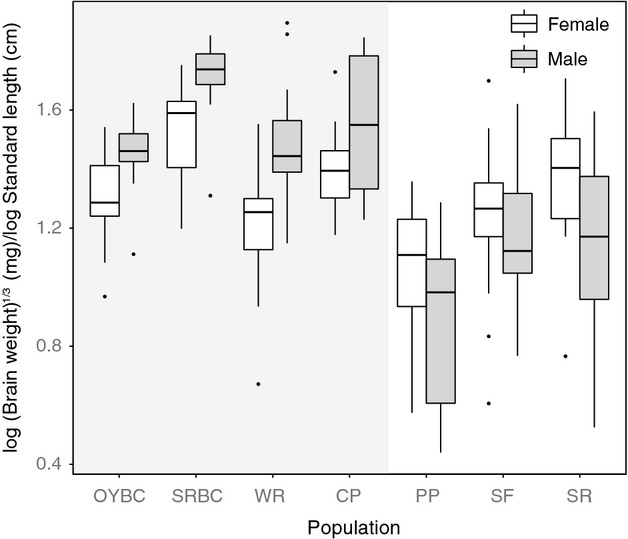
Relative brain weights in seven Canadian populations of threespine sticklebacks. Boxes on the gray background indicate common stickleback populations, whereas plots on the white background indicate white stickleback populations. Population abbreviations are as follows – OY, Oyster Lagoon, BC; SRBC, Salmon River, BC; CP, Captain's Pond, NS; WR, Wright's River, NS; SF, St. Francis Harbour, NS; PP, Porper Pond, NS.

**Figure 2:**
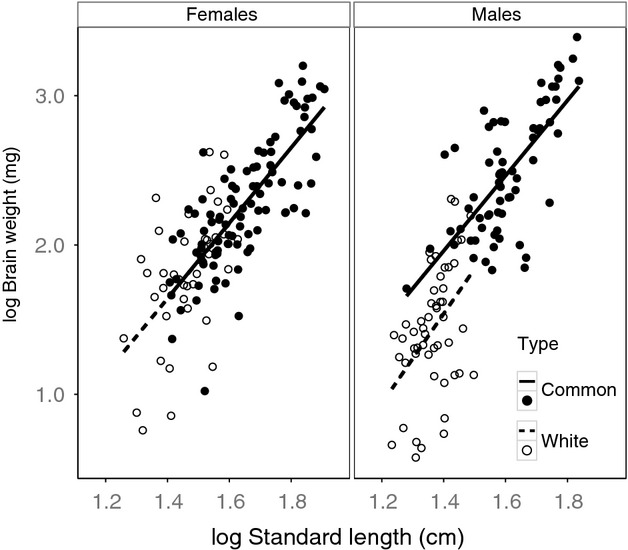
Body weight versus standard length for male and females sticklebacks of two types, white and common, from seven populations in Canada. For clarity, regression lines were determined via standard linear models (no population effect, see text for details on mixed model analysis).

As predicted by the PBH, we found that sexual dimorphism in brain size was altered in white sticklebacks (Table 1, Sex:Type, *F*_1,239_=13.46, *P* = 0.0007). This appears to be driven by a decrease in the intercept of the brain-standard length regression line for male white sticklebacks, rather than a difference in slope (Fig. 2 Males; Table 1, Males: Type, F_1,5_=11.016, *P* = 0.021). In other words, male white sticklebacks have smaller brains than male common sticklebacks across all body sizes. This pattern appears consistent across all three populations of white sticklebacks we sampled (Figs. 1, 2).

**Table 1:** Tests of fixed effects in linear mixed models applied to five threespine stickleback populations (see Table S1 for population identities). “:” denotes an interaction. Bold *P*-values indicate significance at the 0.05 level

	White and common			Males			Females			Common		
				
Source	df	*F*	*P*	df	*F*	*P*	df	*F*	*P*	df	*F*	*P*
SL	1,239	103.9092	**<0.0001**	1,108	71.3239	**<0.0001**	1,126	85.686	**<0.0001**	1,155	47.325	**<0.0001**
Sex	1,239	18.3188	0.1157	–	–	**–**	–	–	**–**	1,155	54.551	**<0.0001**
Type	1,5	6.3784	0.5051	1,5	11.016	**0.0210**	1,5	0.0577	0.8197	–	–	–
SL:Sex	1,239	9.9972	**0.0018**	–	–	–	–	–	–	1,155	0.0422	0.8375
SL:Type	1,239	1.5185	0.2191	1,108	0.1293	0.7199	1,126	0.0992	0.7534	–	–	–
Sex:Type	1,239	13.4630	**0.0007**	–	–	–	–	–	–	–	–	–

When we restricted our analysis to only common sticklebacks, we recovered the same pattern reported by Kotrschal et al. (2012): after accounting for body size, male common sticklebacks have significantly larger brains than female common stickleback (Fig. 1, Table 1, *F*_1,155_ = 54.551, *P* < 0.0001).

## Discussion

Understanding the connection between brain morphology and behavior has long occupied biologists. While contentious, recent data suggest that there is often correspondence between brain size and behavioral repertoire (Gittleman 1994; Farris 2005; Smith et al. 2010). Our results show that male “white” threespine sticklebacks lacking parental care show significantly reduced male brain mass compared with females, the opposite of the pattern found in common stickleback populations. This pattern was consistent across all three white stickleback populations we studied (Fig. 1). These results are in agreement with the expectations of the parental brain hypothesis (PBH) as described by Gonzalez-Voyer et al. (2009a).

In keeping with the PBH, we also showed that the male-biased brain size dimorphism reported by Kotrschal et al. (2012) also exists in Canadian populations. This may thus be a general feature of global (common) threespine stickleback populations.

### Genetic divergence among Nova Scotian populations

Our comparison of genetic divergence between the white and common populations revealed that they are indeed very closely related – common–common *F*_ST_ values are only slightly lower white–common *F*_ST_ values (Table 2) All *F*_ST_ values are comparable to those found between other populations of marine sticklebacks (Leinonen et al. 2006; Hohenlohe et al. 2010). The close genetic relationship between white and common sticklebacks is particularly interesting given that there is evidence of strong reproductive isolation (via assortative mating) between the two types (Blouw and Hagen 1990; Jamieson et al. 1992a). This implies that white and common sticklebacks may be sister species, and likely diverged very recently and/or have experienced on-going gene flow. Although further genetic work is underway, these results suggest that the changes in parental care behavior and brain size we see in the white stickleback likely happened very recently (although demonstrating their direct connect will require further work).

**Table 2:** Pairwise *F*_ST_ values for five Nova Scotian populations of threespine sticklebacks. *F*_ST_ values were calculated based on 12,667 SNPs derived from a GBS dataset (see text for details). Bold values denote *F*_ST_ between types (common vs. white). Values are mirrored above and below the diagonal for ease of comparison

	WR (C)	CP (C)	PP (W)	SF (W)	SR (W)
WR (C)	–	0.0231369	**0.02887124**	**0.03010419**	**0.02941981**
CP (C)	0.0231369	–	**0.03135552**	**0.03248928**	**0.03241964**
PP (W)	**0.02887124**	**0.03135552**	–	0.02247022	0.02119836
SF (W)	**0.03010419**	**0.03248928**	0.02247022	–	0.02289888
SR (W)	**0.02941981**	**0.03241964**	0.02119836	0.02289888	–

Population abbreviations are as follows: CP, Captain's Pond, NS; WR, Wright's River, NS; SF, St. Francis Harbour, NS; PP, Porper Pond, NS. (C) indicates a common population, whereas (W) indicates a white population.

### The parental brain hypothesis in threespine sticklebacks

In their study, Kotrschal et al. (2012) suggest that brain size dimorphism in threespine sticklebacks may be an evolved outcome of the cognitive demands of mate attraction and/or parental care. Our results suggest that parental care may be the larger contributor. This is because while male white sticklebacks perform no parental care, they court much more often than common sticklebacks (Jamieson et al. 1992b). The cognitive demands of courtship are likely at least as high (possibly higher) for the white stickleback compared with common stickleback. Therefore, the cognitive demands of parental care per se appear to better explain extreme sexual dimorphism seen in common sticklebacks and the lack thereof in white sticklebacks.

Nonetheless, it is puzzling that we found a *reversal* of brain size dimorphism, rather than a lack of dimorphism as might be expected under the PBH. Unfortunately, because so little is known about the ecology of white sticklebacks, we can only speculate about why this may be. One possibility is that sexual selection is more potent in white sticklebacks, leading to intensified selection on female brain regions involved in mate choice (Blouw and Hagen 1990; Jacobs 1996; Kotrschal et al. 2012). However, our data do not strictly support this idea, as female white sticklebacks do not appear to show increased brain size compared with female common sticklebacks (Fig. 1). Alternatively, because male white sticklebacks engage in more courtship, they may be investing more energy in gonadal tissue versus brain tissue (i.e., the “expensive tissue hypothesis” as discussed in Aiello and Wheeler 1995; and Pitnick et al. 2006). Testing these hypotheses will ultimately require greater knowledge of the natural history of the white stickleback and more detailed examination of their brain and somatic morphology.

Finally, we recognize that our study (like all others addressing the PBH) is observational and correlative. We do not yet have definitive evidence that parental care behavior *per se* requires a larger brain or that natural selection shapes the evolution of brain size because of its connection to parental care. There are myriad other factors that may explain the difference we observed in brain sexual dimorphism we found in white sticklebacks. That said, given the dearth of studies on the behavioral correlates of brain size in natural populations, we believe our results provide a useful starting point for more detailed investigation of the utility of the PBH for explaining brain size variation.

### Implications for the study of brain evolution

Our results have several interesting implications for the study of brain evolution. First, our genetic data, along with that of Haglund et al. 1990; suggest that the differences in brain size between white and common sticklebacks may have evolved quite rapidly. This is consistent with the results of a recent selection experiment in guppies, which showed a 9% increase in brain size after only two generations of selection (Kotrschal et al. 2013). Interestingly, this contrasts with the findings of Gonzalez-Voyer et al. (2009b,c) who found that brain size has evolved rather slowly during adaptive radiations in cichlids. Thus, there may be a great deal of heterogeneity in the lability of brain size among clades of teleosts.

Secondly, together with previous studies of the PBH in fish (Gonzalez-Voyer et al. 2009a; Kotrschal et al. 2012), our results suggest that social interactions may be an important driver of brain evolution in fish. This broader idea, known as the *social brain hypothesis*, states that the cognitive challenges of social interaction are key selective agents driving brain evolution (Dunbar 1998). This idea is well supported in primates, other mammals, and halictid bees (Dunbar and Shultz 2007; Pérez-Barbería et al. 2007; Smith et al. 2010). While fish do not display the range of social behavior found in these taxa, the social complexity inherent in fish parent–offspring interactions may nonetheless be sufficient to shape brain evolution. Given that parental care is itself known to evolve rapidly in fish (Gross and Sargent 1985), closer examination of the importance of the PBH in these animals is warranted.

### Conclusions and future directions

We found that white sticklebacks display reversed sexual dimorphism in brain size relative to common sticklebacks; white males have smaller brains than white females. We also found general support for the male-biased brain dimorphism in common sticklebacks reported in Kotrschal et al. (2012). These results open the door to a number of exciting future research possibilities. For one, the intercrossability of white and common sticklebacks will allow for the genetic dissection (e.g., QTL studies) of both the loss of parental care and differences in brain morphology. Discovering the genetic basis of these traits will be the key to resolving their biological connection, and ultimately testing the PBH. Secondly, our results motivate more detailed studies of brain morphology in the white stickleback, which will clarify exactly what parts of the brain may be involved in the loss (and normal control) of parental care in threespine sticklebacks.

## Supporting Information

**Figure S1.** Standard lengths of male and female fish from seven Canadian populations of threespine stickleback.

**Figure S2.** Dry brain weights for two Canadian populations of sticklebacks (population information available in Table S1).

**Table S1.** Site information and number of threespine sticklebacks collected at the five locations examined in this study.
