# Supplementary material for: Reversed brain size sexual dimorphism accompanies loss of parental care in white sticklebacks
Source: Ecol Evol. 2014 Jul 27;4(16):3236–43. doi: 10.1002/ece3.1175 (PMC4222210; doi:10.1002/ece3.1175)
Supplement: Supplementary file 3 — Table S1. Site information and number of threespine sticklebacks collected at the five locations examined in this study. [file ece30004-3236-sd3.doc]

**Table S1** Site information and number of threespine sticklebacks collected at the five locations examined in this study. Collections all took place in the early summer of 2012 in British Columbia (BC) and Nova Scotia (NS), Canada.

| **Location** | **Province** | **Type** | **Coordinates** | **Females** | **Males** |
| --- | --- | --- | --- | --- | --- |
| Oyster Lagoon | BC | Common | 49°36'43.44"N, 124° 1'53.70"W | 26 | 8 |
| Salmon River | BC | Common | 45° 21' 9.25"N, -61° 28' 0.38"W | 22 | 15 |
| Wrights River | NS | Common | 45°37'38.52"N, 61°57'56.39"W | 17 | 30 |
| Captain’s Pond | NS | Common | 45°40' 18.79"N, -61°51' 40.42"W | 31 | 13 |
| Porper Pond | NS | White | 45°26'15.51"N, 61°19'26.88"W | 10 | 19 |
| St. Francis Harbour | NS | White | 45°26'36.27"N, 61°18'35.60"W | 21 | 13 |
| Salmon River | NS | White | 45°21'9.25"N, 61°28'0.38"W | 9 | 18 |
